# Evaluation of In-Flow Magnetoresistive Chip Cell—Counter as a Diagnostic Tool

**DOI:** 10.3390/bios9030105

**Published:** 2019-08-31

**Authors:** Manon Giraud, François-Damien Delapierre, Anne Wijkhuisen, Pierre Bonville, Mathieu Thévenin, Gregory Cannies, Marc Plaisance, Elodie Paul, Eric Ezan, Stéphanie Simon, Claude Fermon, Cécile Féraudet-Tarisse, Guénaëlle Jasmin-Lebras

**Affiliations:** 1 SPEC, CEA, CNRS, Université Paris-Saclay, CEA Saclay, CEDEX, 91191 Gif-sur-Yvette, France; 2 Service de Pharmacologie et Immunoanalyse (SPI), Laboratoire d’Etudes et de Recherches en Immunoanalyse, CEA, INRA, Université Paris-Saclay, 91191 Gif-sur-Yvette, France; 3 Direction des Programmes et des Partenariats Publics, Département de la Recherche Fondamentale, CEA, 91191 Gif-sur-Yvette, France

**Keywords:** diagnostic, GMR sensor, whole cell

## Abstract

Inexpensive simple medical devices allowing fast and reliable counting of whole cells are of interest for diagnosis and treatment monitoring. Magnetic-based labs on a chip are one of the possibilities currently studied to address this issue. Giant magnetoresistance (GMR) sensors offer both great sensitivity and device integrability with microfluidics and electronics. When used on a dynamic system, GMR-based biochips are able to detect magnetically labeled individual cells. In this article, a rigorous evaluation of the main characteristics of this magnetic medical device (specificity, sensitivity, time of use and variability) are presented and compared to those of both an ELISA test and a conventional flow cytometer, using an eukaryotic malignant cell line model in physiological conditions (NS1 murine cells in phosphate buffer saline). We describe a proof of specificity of a GMR sensor detection of magnetically labeled cells. The limit of detection of the actual system was shown to be similar to the ELISA one and 10 times higher than the cytometer one.

## 1. Introduction

Routine diagnosis, treatment monitoring and treatment choice would greatly benefit from inexpensive and easy to use versatile devices capable of counting a small amount of whole cells of interest (cancer cells or bacteria) in different matrices [1,2,3,4]. A good example of the lack of adapted screening is breast cancer. Indeed, while it can be cured if detected early, it remains the most lethal cancer for women in developed countries [5].

The flow cytometry, developed since 1965 [6], is the gold standard for whole cell study and labeling. It continues to improve and allows for an increasing number of biological characterizations. The understanding of many biological processes like immune response or cell cycle, the screening of drug efficacy and the enrichment of the data bank about antigen distribution in different cell types are some examples of the huge impact of this technology on the field of fundamental biology [7]. In addition to its wide and growing interest for research purpose, flow cytometry has become routinely used in clinical laboratories over the last 20 years for different pathologies [7,8,9]. This technology is mainly applied in hematology but also in immunology and oncology. For these different disciplines, flow cytometry is used with different purposes like diagnosis, prognosis or treatment monitoring. The main advantages of this technology are the sensitivity and its possibility to measure simultaneously more than 20 parameters per cell [7]. It can also be applied to any cell type from bacteria to circulating tumor cells and to many different biological matrices (blood, bone marrow, solid tissues etc.) [8]. However, this technology still has some drawbacks preventing it from its use for clinical purposes: (i) these devices remain costly, bulky, the data treatment need skilled professionals and cannot be efficiently automated, (ii) the use of fluorescent antibodies makes this technique even more expensive and technically heavy as it necessitates washing steps and (iii) flow cytometry was not developed primarily to count cells but rather to recognize certain cell sub-populations. Some cytometers can give an absolute cell count but discrepancies between devices are common [10].

For early diagnosis purpose and the quantification of disease markers, several other techniques are already available in hospitals and clinical laboratories. Most of detection techniques like mass spectrometry, Western blots or techniques based on Polymerase Chain Reaction require lysis of cells to work with their inner DNA or proteins. In the context of whole cell study however, methods detecting specifically cells by their membrane markers are more relevant. The ELISA test (enzyme-linked immunosorbent assay), largely used, is typically applied to determine the concentration of molecular species in a suspension [11]. This technique is easily transposed to antigens expressed at cell surface as it has been done previously with bacteria [12]. The advantage of ELISA test is its simplicity, high throughput and relative low cost. However, this test still requires trained staff and requires relative long incubation time to reach its nominative performance (for instance the accuracy and reproducibility of the results depend on the reaction time [13]).

Several methods of easy to use cheap sensitive cell counters are being developed by numerous research groups. The development of micro-technologies for biological studies has paved the way for the creation of new devices relying on different detection systems for fast diagnosis. Different solutions are being developed to miniaturize flow cytometers and to simplify its use [14,15]. Optical detection is extremely performant but it requires lasers, precise alignments and interference with some matrices is a common problem either due to their auto-fluorescence or because of solubility issues [16]. Thus, alternative electrochemical or magnetic measurements using simpler detection systems have been proposed and even commercialized [17,18,19,20,21,22]. However these static methods imply washing steps and are conceived for proteins, DNA, RNA or small bacteria detection rather than for eukaryotic cells [23,24,25,26]. Moreover, non-specific interactions remain numerous with this type of devices, leading to reduced sensitivity [27,28]. Taking advantage of the high sensitivity of superconducting quantum interference device (SQUID) or fluxgates, several groups developed static techniques based on magnetorelaxometry [29,30,31]. These tools eliminate the need for washing steps as they discriminate free from bonded magnetic labels. However, SQUID operate at low temperature and their production is quite expensive [32]. Fluxgate-based techniques recently offered proof of their potential by detecting C-reactive protein in serum in 30 min. Still, the electronic circuitry needed for results analyze must be further miniaturized [33]. Other approach are developed in parallel with simpler, less sensitive sensors. Dynamic methods using magnetic detection have been developed first on ferrofluid droplets [34] and raised an increasing interest since Loureiro et al. showed the ability of such devices to detect magnetic objects one by one [35,36] and thus their potential to reach an extremely low detection limit [37]. In addition, the sample can be prepared and tested without any washing step because of the dynamic magnetic detection, insensitive to matrix optical properties [38]. Nevertheless, if washing steps were needed anyway (eg. complex matrices or sample concentration requirements), the system could still offer this possibility as washings can be performed easily with the use of a simple permanent magnet allowing to immobilize beads from the matrix and which is a field-compatible method.

Several methods of magnetic detection have been proposed based on magnetic resonance effect, susceptibility measurements, giant magnetoimpedance (GMI), Hall Effect, Tunnel Magneto Resistance effect (TMR) or Giant Magneto Resistance effect (GMR) [24,39,40,41,42,43,44]. As biological objects are not magnetic and cannot be detected alone using magnetic sensors, the target must first be bound to magnetic particles (MPs or beads). This is possible thanks to antibodies (Abs), whose MPs are coated with, recognizing the target. The very high specificity of antibodies provides an easy way to target precisely the analyte of interest. Moreover the production of polyclonal as well as monoclonal antibodies (mAbs) directed against a given target is now a well-handled procedure in biology labs [45,46]. In a typical magnetic detection process, the mixture of the sample and mAbs-coated MPs is introduced into a microchannel where it flows above the sensors that detect the passage of magnetically labeled biological objects. Several groups worked with GMI sensors, using superparamagnetic particles and Helmoltz coils to generate the AC signal [42,43]. The use of GMR sensors is also a convenient choice for small objects detection due to their high sensitivity and their ease of production [25,39,47,48,49,50]. GMR sensors can now be produced industrially and their size tuned to match the target’s and thus optimize the sensitivity. Moreover, they do not need an AC field to detect the passage of magnetic beads and thus their instrumentation can be simple.

Although several very interesting developments of this technique have been achieved, some difficulties remain [25]. In particular, the binding of the MPs to the target implies mixing the sample with a highly concentrated beads suspension to ensure that the target will meet and bind MPs in a reasonable time. Consequently a lot of free unbound MPs will linger in solution. Moreover, when the target analyte is a living cell, there is inevitably a discrepancy of distribution of the number of magnetic beads bound to each cell. This is due to the natural distribution variation of the number of epitopes per cell recognized by mAbs. Since MPs tend to agglomerate in physiological conditions, the signals created by cells have to be compared not only to those created by single MPs but also to those created by MP aggregates whose sizes depends on the bead type and concentration and on the matrix used. Furthermore, as the signal amplitude depends greatly on the distance between the object and the sensor, it is possible that a small aggregate of beads, moving above the sensor at a short distance gives the same signal as a biological object covered with numerous MPs but flowing further above it. In an attempt to overcome this limit, some ideas have been recently proposed. One consists in using flow focusing to concentrate the detected objects in the bottom half of the channel and avoid this uncertainty [51,52,53]. Yet, while screening tools must remain simple, the use of flow focusing adds a sheath fluid whose flow must be judiciously adjusted. Another idea relying on chip design combining mechanical and magnetophoretic guiding has been proposed to drag all magnetic material at the bottom of the channel without the need of sheath fluid. This method requires precise adaptation to each system and has not been evaluated on any biological model yet [54].

In this work, we suggest a third technique to discriminate specific signals from aggregates, consisting in heightening the floor of the channel above the magnetic sensors so that single beads or small aggregates cannot be detected. We present a complete and reliable process of detection, including negative controls to evaluate specificity, a sensitivity study and a variability evaluation. We have developed a magnetoresistive cell counting device using murine myeloma cells as a biological model. The results have been compared with two standard methods of detection mentioned previously, a microplate sandwich ELISA immunoassay and flow cytometry using the same reagents (mAbs, buffer, samples), which is the only reliable way to compare accurately methods. Similar performances were obtained for the ELISA test and the GMR test while flow cytometry obtained a ten times lower limit of detection.

## 2. Materials and Methods

### 2.1. Sensor Fabrication

The spin valve layers are deposited on a 300 μm thick silicon wafer. The thin films arrangement can be described as follows: Ta(3)/Ni${}_{80}$Fe${}_{20}$(3.5)/Co${}_{90}$Fe${}_{10}$(1.5)/Cu(2.3)/Co${}_{90}$Fe${}_{10}$(2.1)/Ru(0.85)/Co${}_{90}$Fe${}_{10}$(2.0)/Pt${}_{50}$Mn${}_{50}$(18)/Ta(3)/Ru(3) where the thickness of layers is given in nanometers and the target composition is given in percentages. The sensors are then patterned by UV photolithography in a positive resin S1805 and then etched by ion beam etching (IBE). The contact pads are deposited by evaporation of a bilayer Ti(3 nm)/Au(100 nm), after having been designed by photolithography in S1813 positive resin. Finally, a passivation bilayer of 150 nm thick Al${}_{2}$O${}_{3}$ and 150 nm thick Si${}_{3}$N${}_{4}$ are deposited by sputtering on the whole chip surface except on the contact pads. The usual sensor resistance was around 600 Ω. This passivation layer insures a good lifetime of the sensors in aggressive matrices.

### 2.2. Microfluidic Channel Fabrication

The microfluidic channel has been realized by using a classical protocol [55]. A layer of PDMS of an expected thickness of 6 μm is spin-coated (5 min, 2700 rpm, 300 rpm/s) on the sensors after a plasma O${}_{2}$ treatment (15 s, 40 mW, 0.1 mbar) to improve the adhesion. The device is then heated at 110 ${}^{\circ }$C during 20 min and at 60 ${}^{\circ }$C at least for 45 min. In parallel, the 25 μm high and 100 μm wide PDMS channel was molded over an SU-8 mold obtained by UV photolithography and measured by a mechanical profilometer (Alpha-Step, KLA Tencor, Mipitas, CA, USA). After demolding, the injection holes are made in the PDMS using a puncher. After the same aforementioned plasma treatment, the channel is aligned above the sensor using an MJB4 aligner and put in contact with the substrate. The chips are then heated for 20 min at 120 ${}^{\circ }$C and for 1 h at 60 ${}^{\circ }$C.

### 2.3. Cell Culture

Two cell lines were used for the study: first, the NS1, murine myeloma cells, showing an average diameter of 6 μm and expressing at their surface the CD138 protein (Syndecan-1) and second, the Chinese Hamster Ovary cells (CHO) with an average diameter of 10 μm that do not express the CD138 protein. The cell culture media were from Gibco^®^, Life Technologies, Carlsbad, CA, USA.

NS1 cells were cultivated in Dulbecco’s medium with 15% of fœtal bovine serum, 1% of non-essential amino acids, 1% of antibiotics (penicillin and streptomycin) and 1% of L-glutamine at 37 ${}^{\circ }$C under a controlled atmosphere containing 7% of CO${}_{2}$. They were centrifuged at 1000 RPM (centrifuge diameter 344 mm) for 10 min at 9 ${}^{\circ }$C and then diluted in PBS (Dulbecco’s Phosphate Buffer Saline, Gibco, Life Technologies) in which the tests were carried out.

CHO cells were cultivated in Ham F-12 Nutrient Mixture with 10% of fœtal bovine serum, 1% of non-essential amino acids, 1% of antibiotics (penicillin and streptomycin) and 1% of L-glutamine at 37 ${}^{\circ }$C under a control atmosphere containing 5% of CO${}_{2}$. They were washed two times in PBS, let in a solution of 0.25% trypsin-EDTA for 5 min at 37 ${}^{\circ }$C and were centrifuged at 1000 RPM (centrifuge diameter 344 mm) for 5 min at 9 ${}^{\circ }$C. Finally, they were diluted in PBS before use.

### 2.4. Production of IpaD-315 Antibodies

Six to 8-week-old female BALB/c mice were purchased from Janvier Labs, France and maintained in accordance with the French and European regulations on care and protection of laboratory animals (European Community [EC] Directive 86/609, French Law 2001-486, 6 June 2001) and with agreement of the ethical committee (CETEA) no. 15-055 delivered to S. Simon and agreement D-91-272-106 from the Veterinary Inspection Department of Essonne (France). Up to eight mice were kept in each cage and housed in a temperature-regulated-room and had free access to food and water. All animals’ experiments were performed to minimize suffering according to the guideline of the CETEA committee. IpaD-315 murine monoclonal antibody was produced in the LERI laboratory (SPI/CEA Saclay, France). It was raised in BALB/c mice by repeated intranasal immunizations with 20 μg of purified recombinant IpaD protein expressed in E. coli BL21DE(3) [56]. *Ipad* gene was amplified from *Shigella flexneri* (CIP 82.48T) and cloned into the IPTG inducible pET22b(+) vector (Novagen) allowing insertion of a poly-histidine tag sequence at the 3’ end of the gene used for protein purification. Hybridomas were produced by fusing spleen cells of immunized mice with NS1 myeloma cells, according to Köhler and Milstein [45]. IpaD-315 monoclonal antibody was then produced in ascite fluids in BALB/C mouse and further purified by protein A affinity chromatography. The purity of IpaD-315 mAb was assessed by SDS-PAGE in reducing and non-reducing conditions and its isotype determination was performed using Pierce rapid ELISA mouse antibody isotyping kit (Thermo Scientific).

### 2.5. Particle Functionalization

Dynabeads My One Streptavidin T1 were selected. They are 1 μm diameter homogeneous polymer particles embedding superparamagnetic iron oxide nanoparticles. They have been functionalized with two different monoclonal antibodies of the same IgG2a isotype: a rat anti-CD138 mAb (BD Pharmingen) and a murine mAb, IpaD-315 (described in Section 2.4), according to the commercial protocol after their biotinylation and purification.

For mAb biotinylation, 100 μg of antibodies were diluted in 400 μL of 0.1 M borate buffer pH 9.0 containing 6 μL of biotin (Sigma-Aldrich) in DMF at 1 mg/mL and incubated for 30 min at room temperature. Then, 100 μL of 1 M Tris HCl buffer pH 8.0 were added and incubated for 15 min. Finally, the biotinylated mAb was purified from free biotin on Zeba Desalt Spin column (Thermo Scientific) in 0.1 M potassium phosphate buffer pH 7.4 with 0.15 M NaCl. The absorbance of the final solution was measured between 280 and 320 nm to determine the concentration of the purified biotinylated antibody. Biotinylated antibodies were then mixed at room temperature with streptavidin coated beads for 30 min, washed four times in PBS 0.1% BSA and stored in PBS 0.1% BSA at 4 ${}^{\circ }$C until use.

### 2.6. MP Cell Labeling

Several cell concentrations have been used: 10^5^, 3 10^4^, 10^4^, 3 10^3^ and 10^3^ cells/mL while the MP concentration was set to 23 μg/mL corresponding to 2 10^7^ antibodies-coated beads per milliliter. Indeed, the beads concentration must be independent of the cell concentration as this value is unknown in a real sample. In addition to the positive samples with the targeted MP-labeled cells with concentrations described above, three negative samples were prepared and used in experiments: (i) 1 mL of buffer containing only the 23 μg of beads functionalized with anti-CD138 antibody, (ii) 1 mL of buffer containing 10^5^ NS1 cells and 23 μg of the beads functionalized with control IpaD-315 antibody and (iii) 1 mL of buffer containing 10^5^ CHO cells and the 23 μg of beads functionalized with anti-CD138 antibody. Indeed, the detection of typical signals does not mean necessarily that a myeloma cell has been detected: it could also be an aggregate of beads or some MPs bound via non-specific interactions on another kind of cells. A comparison with negative samples is thus needed. The Table 1 summarizes the samples used.

The cells of each sample have been counted at the beginning of each experiment to check the nominal concentration using a Malassez cell. After mixing the MPs with cell suspensions, the samples were incubated at room temperature under a slow rotation for two hours.

### 2.7. Experimental Set-Up

**Figure 1:**
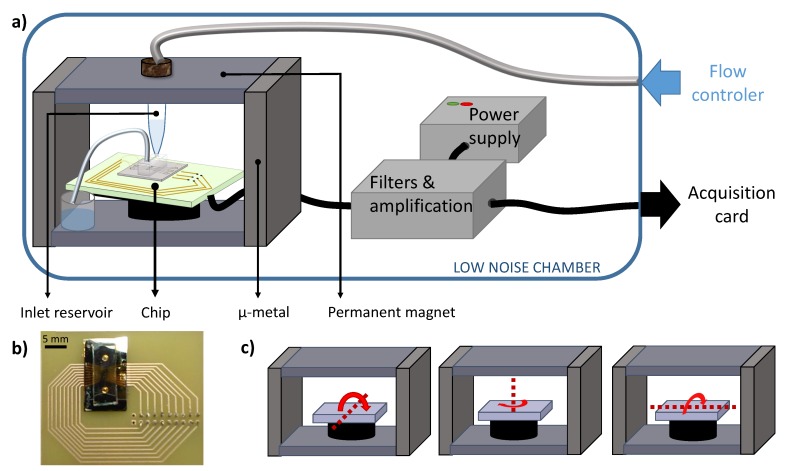
Experimental set-up and data. (**a**) The chip, the reservoir and the collecting vial are inserted in a homogeneous magnetic field. A computer program is controlling the flow using a pressure driver. The applied pressure is set to 300 mbar. Homemade electronic boxes deliver power to the sensor, amplify and filter the signals before sending the data outside the low-noise chamber to the acquisition card. (**b**) Chip photograph. (**c**) Positioning angles.

In an experiment, superparamagnetic objects (labeled cells, unbound MPs and MPs aggregates) magnetized by a permanent magnetic field are flowing above the sensor in a microfluidic channel. The magnetic field must be as homogeneous as possible. Indeed, magnetic gradients, by exerting locally a magnetic force on the particles, can lead to local accumulation of beads in the channel and even clog it. The chips and the inlet and outlet reservoirs are thus inserted in the permanent field created by two ferrite magnets of 3 × 3 × 10 cm^3^ on sides closed with two soft 8 mm iron sheets on top and bottom (see Figure 1a). Using this device, the magnetic field varies by less than 1 mT over the entire surface of the chip (which is 1.5 cm long by 5 mm wide) (see Figure 1b), while the vertical magnetic field reaches 90 mT. The chip is fixed on a support whose angles can be finely tuned to maximize the sensor sensitivity (see Figure 1c). The sensitivity is maximal when the external field is rigorously perpendicular to the sensor surface. At the beginning of each experiment, the position of the sensor is set using a calibrated coil fixed on the magnet which generates a 1 kHz in-plane reference magnetic signal. The aim of this positioning is to maximize the sensitivity and to minimize the noise of the resulting signal. Indeed, the precise location of the sensor influences the random telegraphic noise that appears in some configurations. Then, these two characteristics are measured. The smallest detectable signal, called threshold, is defined as having an amplitude exceeding three times the noise level. In a typical experiment, the sensor noise was evaluated at 50 nT/$\sqrt{\mathrm{Hz}}$, there was 6 μV of noise on the whole bandwidth of 15 kHz and the sensor sensitivity was 2.5 %.mT^−1^.

The device and electronic boxes were used in a magnetically shielded room (2.9 × 2.9 × 2.3 m^3^) made of three μ-metal layers and three aluminum layers. In this environment, the noise level is of 1 nT$\sqrt{\mathrm{Hz}}$ which is low compared to the intrinsic sensor noise. In a real commercial device, a reference GMR sensor (outside of the microfluidic channel) is enough to substract environmental noise, mainly the 50 or 60 Hz magnetic field created by power lines as it has already been done by some groups [57,58].

The flow is driven by a pressure controller (MFCS™-EZ: Microfluidic Flow Control System, Fluigent^®^) and the pressure is set to 300 mbar, typically a sample of 1 mL is flowed in 30 min. The liquid sample is directly injected at the top of the inlet reservoir, made of polyoxymethylene to minimize cells and beads adhesion on its walls. This reservoir is set in vertical position to insure that sedimentation would not impede some cells to go into the channel. The wet part of the reservoir is completely localized in the gap between the two magnets to minimize magnetic forces exerted on the content.

### 2.8. Electronics

**Figure 2:**
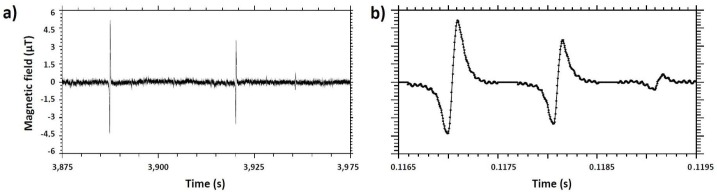
Experimental data. (**a**) Raw experimental recording. (**b**) Recording of the software-selected portions (the same 3 signals are shown).

The electronics is battery supplied to avoid 50 Hz noise. The sensors are biased at voltages between 1 to 2 V and the output signal is amplified 500 times by a low noise preamplifier and filtered at 15 kHz with an additional gain of 20. The signal is then oversampled at 200 kHz using a Data Translation^®^ acquisition card controlled by a homemade software. A schematic view of this set-up is presented in Figure 1a. Then, a homemade software identifies the signals from the total recording and discriminate them from noise artifacts. Numerical parameters were evaluated on a cohort of several thousands of examples from different experiments. For each spotted point above the threshold, the local minimum and maximum are determined by repeatedly incrementing the interval of interest by 15 points until the maximum ($i_{max},V_{max}$) and minimum ($i_{min},V_{min}$) determined are more than 20 points from each edge of the interval. The user determines the direction of signals (k=1 if $i_{max}-i_{min}<0$ and k=−1 if $i_{max}-i_{min}>0$) and the detection threshold ($V_{thr}$). Several checks are then carried out to validate the recording of this peak in the processed file. They must be bipolar ($|V_{min}|>1/3V_{thr}$ and $|V_{max}|>1/3V_{thr}$) with the right orientation ($k(i_{max}-i_{min})<0$), their width ($i_{max}-i_{min}$) must be coherent with the flow velocity (between 25 μs and 2.5 ms) and they must be sufficiently symmetric ($|\frac{V_{max}-V_{min}}{V_{max}+V_{min}}|<0.4$). This last criteria was added to better discriminate signals from radiotelegraphic noise occurring in some experiments. Experimental data before and after treatment are presented respectively in Figure 2a,b.

### 2.9. Comparative ELISA Tests

96 wells plates were coated with anti-CD138 antibody. In each well, 100 μL of a suspension of 10 μg/mL of antibodies in potassium phosphate buffer at 50 mM, pH 7.4 were deposited and incubated overnight at 20 ${}^{\circ }$C. The following day, wells were emptied and filled with 300 μL of EIA buffer (100 mM potassium phosphate buffer pH 7.4 containing 0.1% bovine serum albumin, 0.15 M NaCl and 0.01% sodium azide). The plates were sealed and stored at 4 ${}^{\circ }$C until use.

The day of the experiment, the coated plate was washed once in a washing buffer (50 mM potassium phosphate buffer pH 7.4), 100 μL of serial dilutions of NS1 cells (3 10^6^; 10^6^; 3 10^5^; 10^5^; 3 10^4^; 10^4^; 3 10^3^; 10^3^ cells/mL) in PBS were added per well and incubated under agitation at room temperature for 2 h. Then, the plate was washed three times in the washing buffer and 100 μL of a suspension of biotinylated antibody anti-CD138 at 200 ng/mL in EIA buffer without sodium azide were added per well for a 2h-incubation step under agitation at room temperature. The plate was then washed three times in the washing buffer and 100 μL of a solution of streptavidin conjugated with polymers of horseradish peroxidase (Thermofisher Scientific, Waltham, MA, USA) diluted 15,000 fold in EIA buffer without azide was added into the wells. Finally, after 30 min of incubation under agitation at room temperature, the plate was washed 5 times in the washing buffer and 100 μL of 3,3${}^{\prime }$,5,5${}^{\prime }$-Tetramethylbenzidine (TMB, Thermofisher Scientific) were added per well. After 30 min at room temperature under agitation, 100 μL of 2 M sulfuric acid were added per well and the absorbance of each well was measured at 450 nm (wavelength of absorption of the reaction product) and 620 nm (noise measurement).

The substraction of these two measurements yields the specific signal directly proportional to NS1 cell concentration. The theoretical limit of detection is defined as the lowest cell concentration giving a signal greater than the non-specific binding (mean of eight measurements of EIA buffer) + 3 standard deviations (99.7% confidence). The theoretical limit of quantification is defined as the lowest cell concentration giving a signal greater than the non-specific binding (mean of eight measurements of EIA buffer) + 10 standard deviations (99.9% confidence).

### 2.10. Comparative Flow Cytometry Tests

For flow cytometry analysis, NS1 cells were washed once with PBS/0.5% BSA and 200 μL of serial dilutions of cells (10^5^; 3 10^4^; 10^4^; 3 10^3^; 10^3^ cells/mL) were incubated for 2 h at 4 ${}^{\circ }$C with anti-mouse CD138 labeled with Phycoerythrin (BD Biosciences). After incubation, cells were washed twice with PBS/0.5% BSA and resuspended in 200 μL of PBS/0.5%BSA. The fluorescence was finally assayed for the total volume of 200 μL using a Novocyte flow cytometer (ACEA) and the number of stained cells was evaluated by comparison with cells incubated with buffer alone. Results were analysed using NovoExpress software.

## 3. Results and Discussion

### 3.1. Simulations of Single Magnetic Beads and MP-Labeled Cells

**Figure 3:**
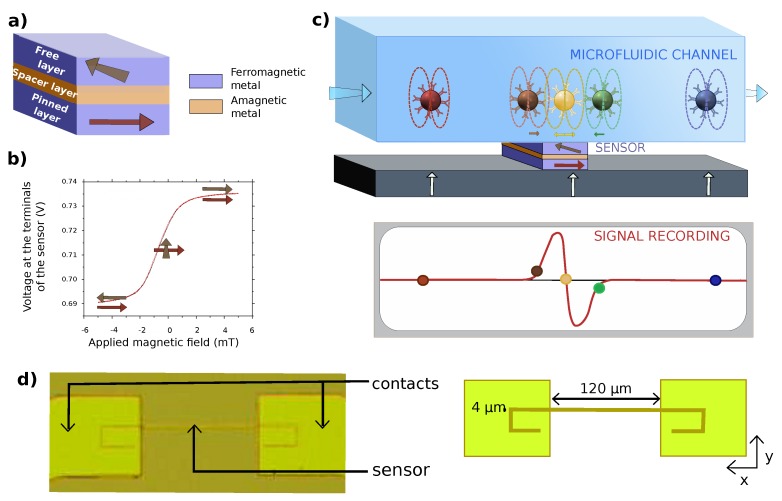
Giant magnetoresistance (GMR) sensor. (**a**) Scheme of the main components of a GMR stack. Free and pinned layers are ferromagnetic and their magnetization are represented by arrows. The spacer is a diamagnetic conductor. (**b**) Experimental sensitivity curve with schematic representation of the relative orientations of the two ferromagnetic layers. This sensor shows a sensitivity of 2 %.mT^−1^ and no hysteresis on its linear portion. (**c**) Schematic of the experiment: Labeled objects are moved by the laminar flow at a given height crossing the sensor at constant speed. The sensor detects variations of the magnetic field due to the induced dipolar field of the beads. The beads are magnetized by a field normal to the sensor plane created by a permanent magnet. (**d**) Photograph and scheme of a processed GMR sensor in yoke shape. The sensor measures 120 μm along the x axis and 4 μm along the y axis.

A GMR sensor is composed of two ferromagnetic metallic layers separated by a nonmagnetic one as shown in Figure 3a. The magnetization of one of these layers is pinned in one direction while the magnetization of the other one is free to rotate in its plane. As the speed of propagation of electrons in a metal strongly depends on the relative orientation of its spin and the magnetization of the metal, this spintronic device will have different properties for spin up and spin down electrons. Depending on the angle between the magnetizations of the two ferromagnetic layers, the overall resistance of the sensor varies as shown in Figure 3b. During the detection process, the MPs themselves are magnetized perpendicularly to the sensor plane by a field created with a permanent magnet and emit a dipolar field. Only the in-plane component of the dipolar field created by the beads is detected by the magnetic sensor since thin-film GMR sensors are insensitive to out-of-plane field variations (z direction) below a critical value. The sensor yoke geometry, designed especially to have just one magnetic domain [59], is shown in Figure 3d and presents a high aspect ratio. This strong asymmetry between the length and width of the device will tend to align all the moments from the free layer according to its length to reduce the magnetostatic energy by moving the two poles created as far as possible from each other. The free layer magnetization is thus along the x axis at zero field, while the other layer is pinned along the y axis. The device is in its most sensitive configuration at zero field and is sensitive only to the y component of the field. Moreover, improving the alignment of moments from the free layer results in a more linear behavior of the sensor. GMR sensors response to small magnetic fields variations are linear on a range of about ±2 mT around zero field (see Figure 3b), which includes the whole range of fields needed for this application.

The situation to be modeled is presented in Figure 3c. Magnetized objects circulate above the sensor in a laminar flow in a microchannel and induce magnetic field variations that are detected by the sensor. Three types of magnetic objects are modeled: single magnetic beads, aggregates of beads and MPs-labeled cells.

The signal corresponding to a single bead moving above the sensor is proportional to the integral over the whole sensor surface of the y-component of the local dipolar field induced at each successive position. For a MP in position $\left(x_{B},y_{B},z_{B}\right)$ with a moment $\vec{\mu }$ making an angle θ with $\vec{z}$ and ϕ with $\vec{x}$ and moving above a sensor of length *L* and width *l*, it is given by the Formula (1) [60].

(1) $$\begin{array}{cc} H_{y} & =\frac{\mu }{Ll}(\left(\frac{y_{l}}{q_{2}^{2}}\left(\frac{x_{r}}{r_{2}}-\frac{x_{l}}{r_{1}}\right)+\frac{y_{r}}{q_{4}^{2}}\left(\frac{x_{l}}{r_{4}}-\frac{x_{r}}{r_{3}}\right)\right)\sin\theta \sin\psi \\ & +(\frac{1}{r_{1}}-\frac{1}{r_{2}}+\frac{1}{r_{3}}-\frac{1}{r_{4}})\sin\theta \cos\psi \\ & +(\frac{h}{q_{2}^{2}}\left(\frac{x_{l}}{r_{1}}-\frac{x_{r}}{r_{2}}\right)+\frac{h}{q_{4}^{2}}\left(\frac{x_{r}}{r_{3}}-\frac{x_{l}}{r_{4}}\right))\cos\theta ) \end{array}$$

where $x_{r},x_{l},y_{r},y_{l},h,r_{1},r_{2},r_{3},r_{4},q_{2}\;\mathrm{and}\;q{}_{4}$ are defined as follows.

$$\begin{array}{cccc} x_{r} & =\frac{L}{2}-x_{B} & x_{l} & =-\frac{L}{2}-x_{B}\\ y_{r} & =\frac{l}{2}-y_{B} & y_{l} & =-\frac{l}{2}-y_{B}\\ h_{\;} & =z_{B}-z_{C}\\ r_{1} & =\sqrt{x_{l}^{2}+y_{l}^{2}+h^{2}} & r_{2} & =\sqrt{x_{r}^{2}+y_{l}^{2}+h^{2}}\\ r_{3} & =\sqrt{x_{r}^{2}+y_{r}^{2}+h^{2}} & r_{4} & =\sqrt{x_{l}^{2}+y_{r}^{2}+h^{2}}\\ q_{2} & =\sqrt{y_{l}^{2}+h^{2}} & q_{4} & =\sqrt{y_{r}^{2}+h^{2}} \end{array}$$

After several simulation tests (data not shown), it has been concluded that the influence of the spatial distribution of the moments in the aggregates was negligible. Signals from aggregates of N beads are simulated as N times the signals coming from a single bead with the same parameters.

**Figure 4:**
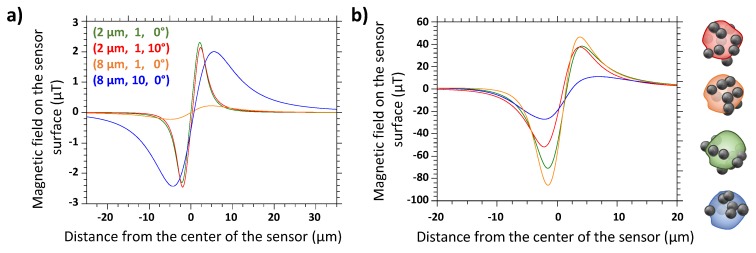
(**a**) Simulation results of the magnetic object detection demonstrating the influence of the three main parameters: distance between object and sensor (Z), number of magnetic particles (MPs) (N) and moment orientation (θ). Curves are labeled by the triplet (Z,N,θ). (**b**) Simulation results for a 6 μm diameter cell at 6 μm height covered by 10 MPs, with four sets of random positions of the beads on the cell surface.

On the contrary, the distribution of MPs on the cell surface was proven to have an influence on the generated signals, as presented in Figure 4b. Cells are thus simulated as spheres with several magnetic beads distributed randomly on their surfaces and with a random angle θ between the direction of their moment and the vertical axis with the constraint of a total magnetization equal to the experimentally measured one (see Section 3.2). This observation leads to the conclusion that detecting one passage with one single sensor cannot be sufficient to deduce precisely the nature and the details of the detected object.

However, our objective is to discriminate the MP-labeled cells from the aggregates of magnetic beads. Three experimental parameters must be chosen together to optimize the discrimination: (i) the chip design, (ii) the permanent magnet, (iii) the magnetic particles. Indeed, with fixed values for these three settings, the values of the two parameters determining the signal shape (the height of the magnetic object and its magnetic moment) are framed. The object distance from the sensor has the largest importance on the amplitude of the signal as shown on Figure 4a. As a consequence, to increase the impact of the number of MPs per object on the resulting signal (value correlated to the nature of this object), the object distance from the sensor needs to be the most homogeneous as possible, hence, the channel must be the smallest possible. This parameter was set to 25 μm, the lowest value at which the channel would not clog after 2 h of use. The permanent magnet must be chosen so that its field is sufficient to have a small average angle θ between the beads magnetic moments and the vertical axis but must be low enough not to pull the pinned layer of the sensor out of plane. This value was set at 90 mT as it was a good compromise knowing the MPs magnetization curves. The choice of the magnetic beads and of the chip design are explained in the following paragraphs.

### 3.2. Deduction of Best Experimental Conditions

The chip design was optimized by testing different configurations of the sensor and microchannel geometries on simulated samples. The main idea was to make some static changes that would not complicate the use of the device but would enhance the discrimination between “positive” and “negative” samples. Samples are called “positive” if they are supposed to contain MPs-labeled cells (they contain a specific complex mAbs-coated beads/cells possessing antigens targeted by the mAbs) and “negative” if they are not, see Table 1.

#### 3.2.1. Sample Characterization

The two kinds of samples had thus to be experimentally characterized to make a proper model. Five sorts of commercial magnetic beads ranging from 200 nm to 2.8 μm in diameter from three companies were tested for the preparation of these samples. Indeed, the choice of MPs is important. Ideal beads should have a magnetic moment sufficiently high to be attracted by a permanent magnet within few minutes to enable simple and quick washing steps (required for mAbs functionalization of MPs before performing the test). They should also have a low saturation field so that a permanent magnet of 90 mT is enough to reach quasi-saturation and, above all, they must present the lowest propensity to aggregation. All these properties were investigated as follows.

First, the magnetic moment of 50 μL of each of the MPs suspensions has been measured using a Vibrating Sample Magnetometer (VSM) and, using the manufacturer number of beads information, the saturation magnetic moment of a single bead has been calculated and the saturation field was determined.

Then, each type of beads was functionalized with antibodies. After this step, their kinetics of cell labeling and their aggregation were studied in parallel. Different concentrations of each type of MPs have been mixed in PBS and with 10^5^ NS1/mL in PBS. For 2 to 3 h, regularly, 100 μL of these suspensions were poured into a well and let to sediment for 15 min. Pictures were then taken under optical microscope. For each cell-containing sample, the distribution of the number of beads per cell was evaluated by visual counting. For the samples without cells, hundreds of these photographs were analyzed with the ImageJ software. They were binarized before the software counted the number of pixels in each aggregate. From these data, the distribution of beads in aggregates was deduced.

**Figure 5:**
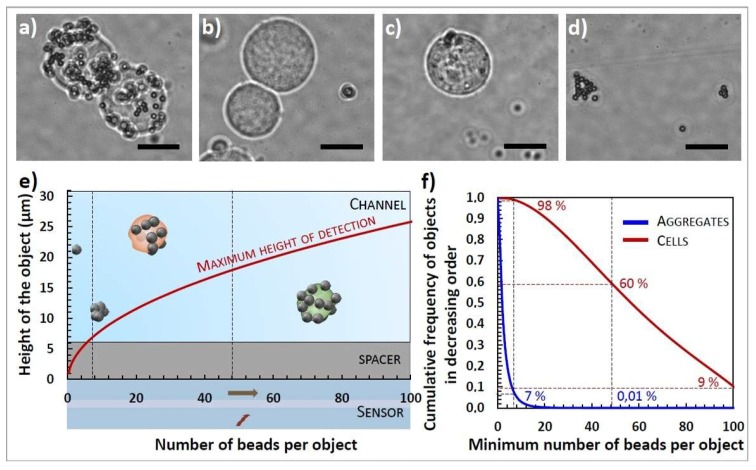
Labeling and aggregation study. Four sample photographs (magnification 100) were taken under optical microscope and illustrate beads repartition. (The scale bars represent 5 μm.) (**a**) Group of two NS1 cells labeled with Dynabeads MyOne functionalized with anti-CD138 mAbs. (**b**) Group of two NS1 cells after two hours-contact with Dynabeads MyOne functionalized with control IpaD-315 mAbs. (**c**) CHO cell after two hours-contact with Dynabeads MyOne functionalized with anti-CD138 mAb (**d**) Dynabeads MyOne functionalized with anti-CD138 mAbs in Phosphate Buffer Saline (PBS). (**e**) Adapted spacer layer thickness estimation. In this illustration, the detectivity is set to 2.2 μT. Relation between the number of beads covering an object and the maximum height at which it can be detected. Objects below the red curve are detectable while objects above are not. (**f**) Corresponding detectable population of cells and aggregates. Graph of the observed cumulative frequency of the number of MPs per NS1 cell and per aggregate in decreasing order. Estimation based on the study of 309 cells in a solution containing 10^5^ NS1/mL and 23 μg/mL anti-CD138 functionalized MPs/mL after 2h-contact and of 18,630 aggregates in a suspension containing 23 μg/mL anti-CD138 in PBS.

Finally, Dynabeads MyOne Streptavidin T1 superparamagnetic have been chosen for the present study (Figure 5, results for the 4 other investigated types of beads can be found in Appendix A). They are 1 μm polymer beads containing maghemite clusters and have a sufficiently high moment that they can be attracted by a permanent magnet within 2 min, thus washing steps (required for mAbs functionalization of MPs) are extremely simple and quick. The saturation magnetic moment of a single bead has been calculated to be 2.1 10^−11^ emu per bead and reached at a field of 700 mT but at 90 mT these beads already have an average magnetic moment of 1.6 10^−11^ emu per bead (data not shown). They are easily functionalized with any kind of purified biotinylated antibodies with high efficiency, with the protocol described in Section 2.5. Moreover the number and size of bead aggregates in the commercial suspension were the lowest of the 5 studied MP types (93% of the objects in the suspension are composed of less than 7 beads, 99% less than 15 beads, the complete curve is presented on Figure 5f) and the distribution of beads per cell was satisfying with an average of roughly 50 MPs per cell (the distribution and the 100 magnification photographs are shown on Figure 5).

#### 3.2.2. Chip Design

The objective is to find the most favorable conditions to discriminate labeled cells from aggregates. As described in details in the Section 3.1, the signals are determined by the dipolar field created by the detected object which depends mainly on the magnetic moment of the object and its height from the sensor. The expression of the dipolar field $\vec{H_{dip}}$ created in B and sensed in C is given by Equation (2) where $\vec{\mu }$ is the magnetic moment of a bead and *N* is the number of beads of the detected object.

(2) $$\begin{array}{c} \vec{H_{dip}}=3\vec{BC}.\frac{\vec{BC}.N\vec{\mu }}{∥\vec{BC}{∥}^{5}}-\frac{N\vec{\mu }}{∥\vec{BC}{∥}^{3}} \end{array}$$

On the Figure 5f, the distributions of beads in cells and in aggregates are given for our system. By using Equation (2), it can be calculated that, between the smallest object in negative samples (1 bead) and the most labeled cell (around 100 beads), the signal is multiplied by 100. Between a height of 1 μm from the sensor and of 10 μm from the sensor, the signal is divided by 1000. Thus, when the channel is directly placed at the top of the sensor, even a signal from a single bead (at 1 μm height) cannot be discriminated from a signal coming from a labeled cell further away from the sensor in the channel, independently of the detection threshold.

Knowing the detectivity of the sensor (experimental characterizations are given on Table 2), the maximal distance from the sensor at which an object composed of N beads can be detected is deduced and plotted in the Figure 5. Above this distance, all the aggregates containing less than N MPs are undetectable. While 98% of NS1 are labeled with more than 7 beads, only 7% of aggregates are composed of more than 7 beads. This minimum number of beads seems to be a good discrimination factor. The Figure 5e shows that objects of 7 beads are undetectable from 6 μm above the sensor.

Adding a separation layer between the sensor and the bottom of the channel eliminates most of the nonspecific signals (from small aggregates and single beads) and improves the discrimination on the number of beads by reducing the importance of the height parameter. Indeed, between 7 and 16 μm height, the signal is divided by approximately 12 only. Without this supplementary layer, objects of small magnetization could still induce large amplitude signals that could be mistaken for labeled cells.

This study leads to the conclusion that the best configuration for our system is the addition of a 6 μm thick separation layer between the sensor and the channel. The thickness of the separation layer needs to be optimized for each couple bead/biological target, because it depends strongly on the expected moment per target and thus on the number of antigens expressed by the target.

### 3.3. Performance of the GMR Chip Test

**Figure 6:**
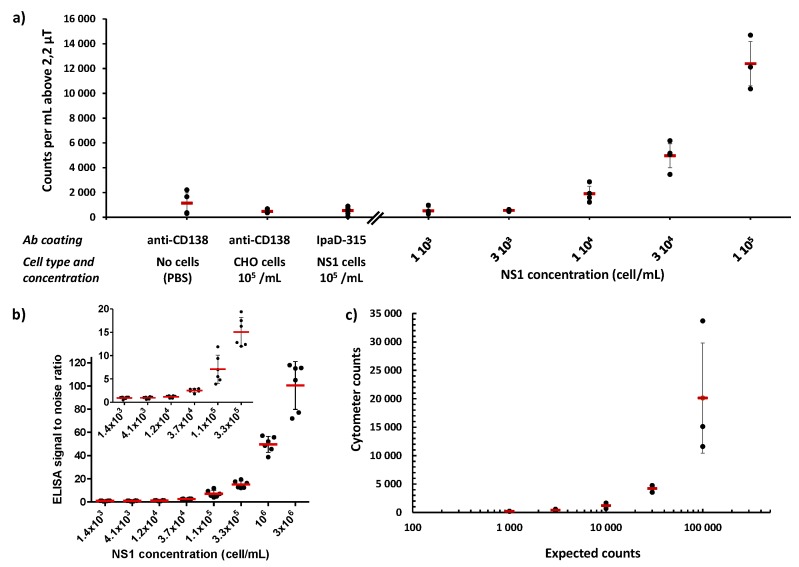
Experimental results of the three tests. All were performed in different days, thus the same samples were not tested with the three techniques. (**a**) Experimental results of the GMR test. Red dashes represent the mean of the experiments. Error bars represent standard deviations from the experiments. (**b**) Experimental results of the ELISA tests. Different concentrations of NS1 cells in PBS were detected using the homologous sandwich ELISA involving anti-CD138 mAb as capture and tracer antibody in a 5 h sequential format. The signal to noise ratio was calculated from the mean of eight measurements of PBS alone. Red dashes represent the mean of the three independent experiments, each performed in duplicate. Error bars represent standard deviations from the three experiments. The insert shows the low concentration part of the curve. (**c**) Experimental results of flow cytometry presented as counts per milliliter for each concentration. Red dashes represent the mean of the three independent experiments. Error bars represent standard deviations from the three experiments.

Seven experiments (See Table 2 and Figure 6a) were realized on six days using 4 similar chips with different sample volumes ranging from 200 to 400 μL and the number of events was normalized to a volume of 1 mL. To avoid biased results, samples have been injected in the chip in different orders at each experiment and between two samples the chip was washed with deionized water and dried. For each experiment, the sensitivity and the noise level of the sensor have been measured and the detection thresholds were deduced (given in Table 2). In order to coherently treat data from these seven experiments, only signals above the highest detection threshold (2.2 μT) were considered.

Between 60 and 2200 counts per milliliter were found in each type of negative control samples in PBS (14 measurements in total). Studying these results allow us to determine a count threshold characterizing the test. The count threshold above which the sample can be considered positive (the detection threshold) is calculated as the average of the values obtained for the negative test having the most counts (thus, the anti-CD138 beads) added to three times their standard deviation. This method concludes to an average non-specific count of 1.1 10^3^ counts per milliliter and a detection count threshold of 3.6 10^3^ counts per milliliter.

Looking at the positive sample results, this threshold corresponds to a concentration between 1 and 3 10^4^ NS1 per milliliter.

### 3.4. ELISA Test Sensitivity

The ELISA test was repeated in three independent experiments in order to compare its detection limit to the one of the GMR sensor. Results are presented in Figure 6b. For this 5 h-test in PBS suspensions, the detection limit (the lowest concentration at which the test can determine positivity of the sample) was found equal to 2.0 10^4^ NS1/mL ± 1.8 10^4^ while the quantification limit (the lowest concentration at which the test can give a correct estimation of the sample concentration) was found at 6.7 10^4^ NS1/mL ± 2.9 10^4^.

### 3.5. Flow Cytometry Results

The number of NS1 cells was evaluated by flow cytometry using a specific monoclonal antibody against the CD138 surface molecule. The living cells were selected by size and cell granularity and the number of CD138+ cells in each sample was evaluated by comparison with cells incubated with buffer alone. As can be seen in Figure 6c, the evaluation of the number of positive cells for the CD138 marker is possible up to a cell concentration in the sample equal to 3 10^3^ cells/mL.

## 4. Conclusions

In this article, the different steps of the conception of a magnetoresistive chip cell-counter were detailed. This detection technique has a great potential. The production, use and integrability of GMR sensors are easy and the tool allows for the detection of targets one by one. This test was evaluated regarding several essential qualities of diagnostic tools (sensitivity, specificity, reproducibility and duration) on a biological model, murine myeloma cells immunocaptured by commercial magnetic beads of 1 μm in diameter. The reached sensitivity of about 10^4^ cells/mL is equivalent to that of an ELISA test realized with the same reagents (NS1 cells, mAbs, buffer ...). Our test is simpler to perform than an ELISA test. Indeed, the GMR test can be performed within 2h30 (2 h of labeling as assessed by our kinetic study, briefly described in Appendix A and 30 min/mL of sample for the test) without any washing steps, while the compared ELISA test requires several washing steps. Data treatment can be done in a few minutes for ELISA test and can be integrated in the acquisition chain and done in real time for the GMR test. One can note that both techniques can benefit from large parallelization of tests. Moreover, the time of the GMR test can be further reduced by increasing the flow rate in the channel. The labelling processes strongly depends on the target, the beads and the biological probe and other groups reported times between 30 and 180 min [51,53,57]. This time will have to be optimized on the final system, in the real biological sample.

**Figure 7:**
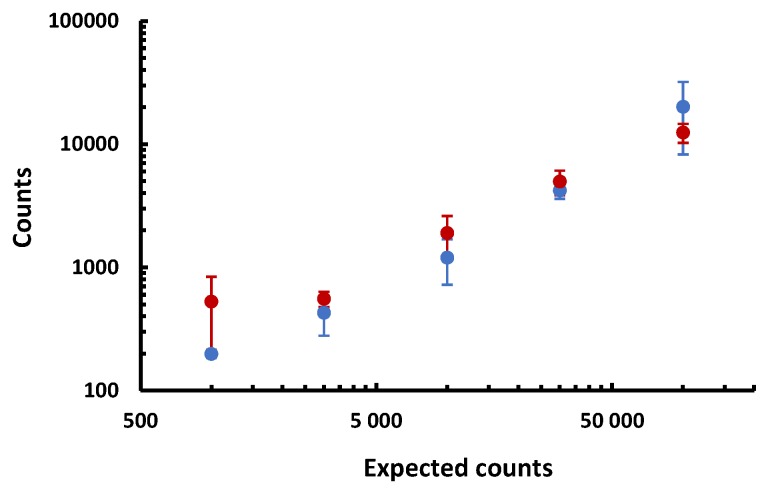
Experimental results of flow cytometry (in blue) compared with experimental results of the GMR test (in red). Mean values and standard deviations are represented.

Flow cytometry, although not optimized to give absolute cell counts, have a sensitivity ten times lower than the GMR test. However, this method is more complicated, with washing steps, causing loss of cells and thus discrepancies in counts. The Figure 7 shows the extrapolated number of cells counted by both techniques in positive samples of 1 mL as a function of the expected counts. The agreement between the two techniques is remarkably good for all concentrations except at 10^3^ NS1/mL. Our technique however, presents the interest that the count of signals can be automated while flow cytometry data treatment requires an expert.

The relatively high limit of detection of some 10^4^ cells/mL is due to two main phenomena. First, some specific events are missed. Indeed, some less efficiently labeled cells are flowing high in the channel and cannot be detected specifically. Secondly, the detection count threshold has a high value. This can be explained both by the number of beads aggregates, increasing the average number of non-specific signals and by the variability of experimental parameters, increasing the standard deviation of the number of non-specific signals. These uncertainties rise from the use of 5 distinct batches of functionalized beads for the experiment, the random order in which the samples were passed and the involuntarily fluctuations in channel geometry.

This study shows the importance to take into account the biological parameters (antigen distribution, labeling efficiency, cell survival, matrix effect, etc.) in the test evaluation. The high detection count threshold value demonstrates the crucial importance of having negative controls and to repeat experiments in different conditions several times in order to define correctly performances of such technologies. The development of diagnostic tests are based on these two pillars (physical and biological parameters) and correct definitions of performances of a test should systematically integrate these cross-cutting aspects. Here, the focus was set on a rigorous evaluation of non-specific signals measured by the GMR sensor. The study showed that these non-specific signals were due to the detection of beads aggregates.

To lower the detection threshold without complicating the device, the challenge is to diminish drastically the number and the sizes of the MP aggregates. As a matter of fact, decreasing the number of beads in aggregates would enable great changes in the chip design. The separation layer, added to reduce the impact of non-specific events, could be thinned and thus less efficiently labeled cells would be easier to detect. A better understanding of these aggregation phenomena and development of solutions to reduce the number of these non-specific events will help to reach a better reproducibility and sensitivity.

The elimination of aggregates can be performed by microfluidics sorting techniques relying on hydrodynamic or magnetodynamic forces [61,62,63,64,65] but this would necessarily waste a certain amount of expensive mAbs-coated beads. Another way to deal with these beads suspensions instabilities would be to address directly the cause by a better design of the magnetic beads, such as adding a PEG coating [66].

The real solution may lie in designing magnetic beads tuned especially for this application and thus, to continue the development of this diagnosis tool, the natural next step should be to add chemistry as a third project pillar.

## Figures and Tables

**Table 1:** Summary of used samples.

Sample Type	Cells/mL	Antibody Beads Coating
	1 × 10^5^ NS1	anti-CD138
	3 × 10^4^ NS1	anti-CD138
Positive	1 × 10^4^ NS1	anti-CD138
	3 × 10^3^ NS1	anti-CD138
	1 × 10^3^ NS1	anti-CD138
	1 × 10^5^ NS1	IpaD315
Negative	1 × 10^5^ CHO	anti-CD138
	No cell	anti-CD138

**Table 2:** Experimental conditions of the seven experiments. The threshold of each experiment is defined as the lowest detectable signal (with a signal to noise ratio at 3). Results are given as a number of events above 2.2 μT detected per milliliter of sample. The average count is given with its standard deviation (SD) for each sample. Control samples are presented at the bottom of the table, separated from positive samples. The highest count in negative samples, in bold, is obtained for anti-CD138 beads in PBS for which the average value added to three standard deviations gives 3.6 10^3^ counts.

		Day 1	Day 2	Day 3	Day 4	Day 5	Day 6		Summary
Sensor		Sensor A		Sensor B	Sensor C		Sensor D		Different
Separation layer thickness		6.2 μm		6.4 μm	5.7 μm		5.5 μm		devices,
Channel height		26.5 μm		27.4 μm	25.3 μm		23.1 μm		samples and
Beads batch		1	2	3	4	5	5		conditions.
Threshold (μT)		1.6	1.8	2.2	0.47	0.46	0.48		Same
Sample volume (μL)		400	200	300	300	300	300	300	experimenters
Cells	mAbs	Counts of signals above 2.2 microteslas per milliliter							Average ± SD
10^5^ NS1	anti-CD138	12,123	14,700	10,367					1.2 × 10^4^ ± 1.8 10^3^
3 10^4^ NS1	anti-CD138				3463	5173	6180	5070	5.0 × 10^3^ ± 9.7 10^2^
10^4^ NS1	anti-CD138				2867	1223	1927	1597	1.9 × 10^3^ ± 6.1 10^2^
3 10^3^ NS1	anti-CD138				467	630	517	607	5.6 × 10^2^ ± 6.6 10^1^
10^3^ NS1	anti-CD138				500	977	280	353	5.3 × 10^2^ ± 2.7 10^2^
10^5^ NS1	IpaD315	895	60	660	637	723	313	520	5.4 × 10^2^ ± 2.6 10^2^
10^5^ CHO	anti-CD138					690	380	367	4.8 × 10^2^ ± 1.5 10^2^
∅	anti-CD138	1665	375	2213	310				**1.1 × 10^3^ ± 8.2 10^2^**

**Figure A1:**
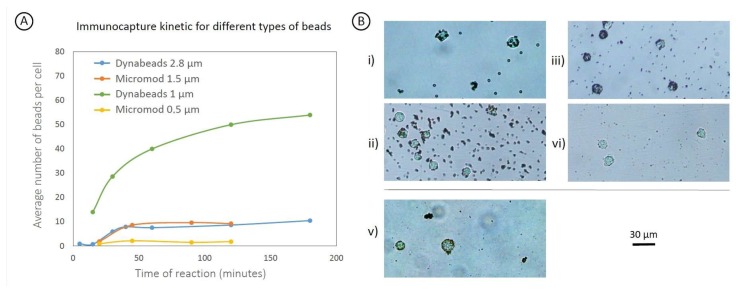
(**A**) Kinetic of NS1 labeling for 4 types of microbeads. (**B**) 20 magnification photographs of NS1 cells incubated 2 h with: (i) Dynabeads 2.8 μm (ii) Micromod 1.5 μm (iii) Dynabeads 1 μm (iv) Micromod 0.5 μm (v) Adembeads 200 nm.

## Abbreviations

The following abbreviations are used in this manuscript:

BSA	Bovine Serum Albumine
CHO	Chineese Hamster Ovary cells
ELISA	Enzyme-Linked ImmunoSorbent Assay
GMR	Giant Magneto-Resistance
mAb	Monoclonal antibody
MP	Magnetic Particle
PBS	Phosphate buffer saline
PDMS	Polydimethylsiloxane

## Appendix A. Magnetic Particles Aggregation Study

mAbs-functionalized 200 nm carboxyl-Adembeads (Ademtech^®^), 500 nm streptavidin nanomag^®^-D (Micromod), 1 μm Dynabeads^TM^ MyOne^TM^ Streptavidin T1 (Invitrogen^TM^), 1.5 μm streptavidin sicastar®-M (Micromod) and 2.8 μm Dynabeads^TM^ M-280 Tosylactivated (Invitrogen^TM^) suspensions were analyzed in terms of aggregate distribution (data not shown). Then, cell immunocapture kinetic studies were realized with an optical microscope. Data from 200 nm beads were hardly exploited but it was clear that beads aggregated considerably while no excessive labeling was visible at cell surface (Figure A1B(v)). The other 4 types of MPs gave quantified results presented on Figure A1 as plots of average number of beads per cell against time. The 500 nm-diameter Micromod beads labelled poorly the cells after 2 h. 1.5 μm diameter Micromod beads labelled the cells correctly but aggregates were far too numerous for those beads to be of any interest. The best options were the two kinds of Dynabeads^TM^. The estimation of number and size of aggregates for these beads showed that 1 μm beads seemed to form labeled cells more distinguishable from beads aggregates than the 2.8 ones. However, to confirm that, there were attempts to decrease the number of aggregates for 2.8 μm beads as they reached the maximum cell labeling quicker. They were sonicated for 30 s to 1 minute for 1 to 3 times before use with no conclusive results. Then, they were vortexed and sonicated alternatively 3 times with cycles of 2 and 1 min respectively. Finally they were added to several detergent alone or in combinations at 0.1% each (SDS (sodium dodecyl sulfate), tween 20, triton X-100, CHAPS (3-[(3-cholamidopropyl)dimethylammonio]-1-propanesulfonate)) either in PBS or in deionized water before being submitted to the 3 cycles of sonication-vortices described above. While most of these attempts remained unsuccessful, the suspension of beads in deionized water containing all detergents and the suspension in deionized water containing SDS and tween 20 showed significant improvements: the percentage of single beads improved from 66% in the suspension in PBS without detergent to 90% and 84% respectively. Once this observation was done, the cell survival was evaluated in the presence of different detergent concentrations ranging from 0.01% to 1%. All cells were lysed after 2 h contact with all these detergent concentrations. Dynabeads^TM^ of 1 μm were thus the optimum and detergent use was abandoned. Sonication and vortex did not improve their aggregation.
